# Sadistic Sexual Crimes Against Children: Comparing the Manifestation of Sexual Sadism and Crime-Commission Process

**DOI:** 10.1177/0306624X221132225

**Published:** 2022-11-15

**Authors:** Kylie S. Reale, Julien Chopin, Alexandre Gauthier, Eric Beauregard

**Affiliations:** 1 Simon Fraser University, Burnaby, BC, Canada; 2 Laval University, Québec, QC, Canada; 3 University of Montréal, QC, Canada

**Keywords:** sexual sadism, SeSas, child victims, comparison, dimensions

## Abstract

Individuals who commit sexual offenses against children have been shown to be distinctive from adult offenders across both individual and crime characteristics. However, an examination of the literature shows that there are gaps in the research related to whether sadism manifests differently between those who target child compared to adult victims. The current study therefore aimed to explore the differences in the characteristics, crime-commission process, and the dimensions of sadism between sadistic crimes of children (*n* = 101) compared to those of adults (*n* = 433). Sexual sadism was assessed with the Sexual Sadism Scale (SeSaS) and binary logistic regression analysis and multidimensional scale analysis (MDS) were performed to examine differences between these two groups. Our results showed that sadistic fantasies manifested into four dimensions for both adult and child victims. In terms of differences, sadistic sexual offenses involving children appear to be reflective of deviant fantasies related to overlapping paraphilias (e.g., sadism and pedophilia). Conversely, sadistic crimes involving adult victims involve a crime-commission process that involves a greater degree of structure to control their adult victim and decrease their risk of identification. Implications for clinical assessment and police investigations are discussed.

Krafft-Ebing (1886/1998) popularized the concept of sadism in his book *Psychopathia Sexualis*, which he defined sadism as the experience of pleasure caused by acts of cruelty and corporal punishment that can also involve the desire to humiliate, hurt, hit, or even destroy others to experience sexual pleasure. Although there is no agreed definition of sexual sadism, there is empirical consensus that is that it is not distinguished by violence, but rather, by the humiliation, degradation, subjugation, and suffering of the victim, which makes the person feel powerful and sexually aroused (Healey et al., 2013). Although there has been considerable research on sadistic sexual offenses (see Longpré et al., 2019 for a review), empirical knowledge on the differences between individuals who commit sadistic sexual offenses against adult and child victims remains very limited. Nonetheless, there is some evidence to suggest that sadistic offenses involving children involve a different crime-commission process (e.g., Chopin et al., 2022) and may also manifest into different behavioral dimensions and may therefore vary in severity, intensity, and deviant sexual fantasies (e.g., Chopin & Beauregard, 2023; Knight et al., 1989). The current study therefore aims to provide the first empirical examination of the differences in the behavioral manifestations and crime-commission process of sadistic sexual crimes of adults and children to help improve the assessment of sexual sadism (e.g., Mokros et al., 2012; Nitschke et al., 2013; Stefanska et al., 2019) as well as the investigation of these crimes (e.g., Chopin et al., 2022; Reale et al., 2020).

## Literature Review

### Sexual Sadism: A Dimensional Approach

Sexual sadism has been included in the *Diagnostic and Statistical Manual of Mental Disorders* (DSM-5; American Psychiatric Association, 2013) since the mid-twentieth century. In it, sexual sadism is defined as “pleasure and sexual arousal that is rooted in fantasized or actual infliction of psychological or physical suffering on a victim”. Since the third edition of the DSM (American Psychiatric Association, 1980), the diagnosis of sexual sadism requires the fantasies or behaviors must be severe, recurrent, and last for a minimum period of 6 months. Moreover, the behaviors must be directed toward non-consenting partners, or the sexual urges or fantasies must cause marked distress or interpersonal difficulties. Although the DSM-5 is the most widely used diagnostic tool for the assessment of sexual sadism, among researchers and clinicians, there has been considerable debate surrounding the required behavioral criteria, the elements that are necessary for sexual excitement, and the severity of the cut-off point (see Longpré et al., 2018 for a review). As a result, the diagnosis of sexual sadism has been criticized for lacking reliability and providing inaccurate assessments of sexual sadism (e.g., Longpré et al., 2019; Marshall, Kennedy & Yates, 2002, Marshall, Kennedy, Yates & Serran, 2002; Nitschke et al., 2013; Stefanska et al., 2019). This has also led to challenges in estimating the true prevalence of this disorder with studies estimates ranges from 5 to 50% of all sexual offenders, and 35% of sexual homicides (Chopin et al., 2022).

As a result of these inconsistencies, several researchers have argued that sadism should be viewed dimensionally (e.g., Chopin & Beauregard, 2022; Longpré et al., 2018; Marshall & Hucker, 2006; Mokros et al., 2012). This approach suggests that sexual sadism should be defined and assessed based on severity (e.g., low, moderate, high), rather than dichotomously (i.e., presence vs. absence) (Longpré et al., 2018, 2019; Nitschke et al., 2013). Consistent with a dimensional approach, researchers have developed psychometric assessment instruments for sexual sadism that are composed of behavioral indicators rated through crime-scene information. One of the most widely validated and reliable tools is the *Severe Sexual Sadism Scale* (SeSaS; Nitschke et al., 2009). This 11-item behavioral scale uses a cut-off score of 4 to differentiate severe sadists from non-sadists and has been shown to have the potential to improve the validity and reliability of the diagnosis of sexual sadism (Gonçalves et al., 2020).Interestingly, recent studies have shown that the manifestation of sadistic fantasies through crime scene behaviors was part of a continuum of severity, with certain acts associated with more or less serious crimes (Chopin et al., 2021; Mokros et al., 2012; Nitschke et al., 2009; Reale et al., 2017). For example, studies using the SeSas have shown that sexual mutilation and foreign object insertion can be associated with homicide, (Mokros et al., 2012; Nitschke et al., 2009).

### Differences in the Manifestation of Sadism in Crimes Involving Adults Versus Child Victims

There is some evidence in the literature to suggest that the manifestation of sadistic fantasies and level of intensity may differ between sadistic sexual offenses involving adults compared to children, however, no study has explicitly tested this assumption. For example, Chopin et al. (2021) examined the heterogeneity in the manifestations of sexual sadism involving a mixed sample of adult and child victims of sadistic sexual homicide. They identified four different classes of sadistic sexual homicide: 1) individuals manifesting their sadism with sexual acts centered on anal and oral penetration, 2) those centered on inserting foreign objects into the victim’s orifices, 3) those who collect trophies belonging to the victim, and 4) those who torture and mutilate their victims. Interestingly, this study found that the torture/mutilation class was the most likely to target children, indicating that sadistic sexual offenses against children may be characterized by different behavioral manifestations. In a study by Knight et al. (1989) which focused exclusively on a sample of individuals who committed child molestation, two sadistic subtypes were observed. The first involved the *sadistic type—high injury*, which was characterized by sexual arousal or pleasure from placing the victim in pain or fear. Behavioral indicators for this subtype included the use of violence to facilitate sexual arousal, or the presence of bizarre, ritualized, and unusual acts in addition to aggressive sodomy or foreign object insertion. The second sub-type with the *sadistic—muted*, which was characterized by the presence of object insertion without injury, and/or reported or behavioral evidence of sadistic sexual fantasies (e.g., bondage, humiliation, or bizarre and unusual acts) or sodomy.

More recently, Chopin and Beauregard (2022) found that sexual domination is present in all cases of their sample of sadistic sexual homicide of children, however, degrading and humiliating behaviors were entirely absent. This findings highlights the utility of examining behavioral indicators of sexual sadism through crime scene behaviors considering that the DSM diagnosis of sexual sadism requires that an individual is sexually aroused by physical or psychological suffering (i.e., humiliation) of their victim (DSM-5; American Psychiatric Association, 2013) and several studies have identified humiliation as a main method of sexual arousal in sadistic sexual acts (e.g., Healey et al., 2013; Marshall, Kennedy, Yates & Serran, 2002; Richards & Jackson, 2011). Moreover, this suggests that sadistic fantasies and the level of intensity in sadistic sexual behaviors may be different in individuals who victimize children compared to adults. Thus, by examining the behavioral indicators of sexual sadism it may be possible to aid in a more accurate assessment of sexual sadism, (Mokros et al., 2012; Nitschke et al., 2013) as well as provide new insights into differences in underlying sexual fantasies between individuals with child or adult victims. This is especially relevant for individuals who are likely motivated to deny or downplay their sadistic urges or fantasies (Stefanska et al., 2019).

#### Offender and crime characteristics

Another important practical area of research in sadistic sexual crimes is related to improving investigative efforts. In particular, researchers have examined the crime scene behaviors involved in sadistic crimes (pre-crime, crime, and post-crime) to help identify behavioral markers of sexual sadism that can be used to help prioritize suspects, identify underlying motivations, and provide insight into the situational contexts to the crime (Chopin et al., 2022; Dietz et al., 1990; Reale et al., 2020). A key finding that has emerged in the examination of sadistic sexual crimes is that these offenses are typically characterized by behaviors that are indicative of detection avoidance strategies. In particular, studies have shown that sadistic crimes are highly structured and planned. For example, sadistic offenses have been found to involve greater forensic awareness (e.g., destroying and removing evidence; Dietz et al., 1990; Reale et al., 2017, 2020), are more likely to involve transporting or hiding the victim’s body to prevent detection (Beauregard & Proulx, 2002; Reale et al., 2020), and are located in isolated areas that are preselected by the offender to reduce their risk of detection (Dietz et al., 1990; Reale et al., 2020). To our knowledge, only one study by Chopin and Beauregard (2022) compared the crime-commission process of sadistic sexual homicides against child victims to non-sadistic sexual homicide. They observed that sadistic sexual homicides against children only involved partial forensic awareness, in that offenders’ chose deserted locations to assault and dump the victim’s body, used strategies to limit the victim’s resistance, but did not destroy or remove forensic awareness or protect their identity. Greater insight into differences in the individual and crime characteristics of sadistic offenses involving adult and child victims can help police investigative efforts to prioritize suspects and inform investigative strategies when the offender is unknown (Chopin et al., 2022; Reale et al., 2020).

## Current Study

An examination of the literature shows that there are important empirical gaps related to whether sadism manifests differently between those who target child compared to adult victims (Chopin & Beauregard, 2022; Chopin et al., 2021; Knight et al., 1989). Identifying differences in the manifestation of sexual sadism between adult and child offenders can improve our understanding of sadism and also aid clinicians in reaching more accurate diagnoses as well as improve the investigation of these offenses. Considering that lack of empirical research which compares these two victim-age groups, this study takes an exploratory approach to address the following research questions:

(1) Are the dimensions (i.e., behavioral manifestations) of sadism different when the victims are children?
(2) Do individuals involved in sadistic crimes of children exhibit different characteristics than those targeting adults?
(3) Does the crime-commission process adopted by individuals involved in sadistic crimes of children differ from those of adults?

## Methodology

### Sample

The sample used in this study includes 101 individuals involved in child sexual abuse (i.e., 15 years old and less, see Chopin & Beauregard, 2023; Leclerc et al., 2011), for which sadism was assessed with the SeSaS (i.e., a score of at least 4, see Nitschke et al., 2009). This sample comes from a larger national database of 735 male sadistic sexual offenders involved in sexual assaults, which occurred between 1990 and 2018 in France. This database includes information on offender, victim, and crime characteristics and data are derived from various sources of information. To avoid missing data, information is compiled by a team of crime analyst experts on violent crimes for operational purpose. Although it is still possible to have missing values as the information may not always be known, this was not the case with the variables examined in this study. For each case, the information comes from investigative reports, interview reports, medical/autopsy reports provided by pathologists, psychological reports provided by a team of forensic psychologists, and crime scene forensic reports.

In order to compare sadistic crimes involving child victims with those involving adult victims, we selected a sample from the same database of 443 individuals who had committed sadistic sexual crimes against adult victims. Similar to the selection of cases involving child victims, we selected only those cases for which the SeSaS score was at least 4 (Nitschke et al., 2009). Regarding the operationalization of an adult victim, we decided to restrict the adult victims to those aged 21 years old or more (i.e., 21 years old is the age of majority in the United States and is often used as the cut-off to designate adults). This decision was made to avoid including cases that could fall in between the two categories, thus introducing noise into the analyses and findings. Finally, no cases involving both adult and child victims were included in our sample.

### Sexual Sadism Assessment

Sexual sadism was assessed with the Sexual Sadism Scale (SeSaS) which was chosen because previous studies indicated that it was strongly related to the clinical diagnosis of sexual sadism (Longpré et al., 2018; Mokros et al., 2012; Nitschke et al., 2009, 2013).^1^ Consistent with to most studies using the SeSaS to assess sexual sadism, we used only Part 1 (e.g., Chopin & Beauregard, 2021; Chopin et al., 2022; Eher et al., 2016; Gonçalves et al., 2020). Part 1 of the SeSaS is composed of 11 items, including: 1) individual is sexually aroused by the act (i.e., information comes from interviews with individuals involved in crimes, victim statement in cases of non-lethal outcome, and crime scene reports denoting traces of sexual conduct such as used condoms or semen; see Nitschke et al., 2009); 2) individual exercises power/control/domination over the victim; 3) individual humiliates or degrades the victim (i.e., defined as reducing the victim to a lower position, demeaning the victim to a low, destitute, or demoralized state. Humiliation or degradation can be achieved verbally or through particular actions such as forcing the victim to crawl, urinating on the victim, defecating on the victim, see Nitschke et al., 2009); 4) individual tortures the victim or engages in acts of cruelty (i.e., defined as the infliction of intense pain and suffering with behaviors such as piercing, pinching, pulling, biting body parts, see Nitschke et al., 2009); 5) individual mutilates sexual parts of the victim’s body (mutilations were committed ante mortem and none was committed only after the death of the victim, but they may have led to the death of the victim and be continued after the victim’s death); 6) individual engages in gratuitous violence toward the victim (i.e., was coded as present if the amount of violence clearly exceeded the degree of force that would have been necessary to merely control the victim. For example, behaviors such as use of excessive physical violence, inflicting wounds, see Nitschke et al., 2009); 7) individual keeps trophies of the victim; 8) individual mutilates nonsexual parts of the victim’s body (mutilations were committed ante mortem and none was committed only after the death of the victim, but they may have led to the death of the victim and be continued after the victim’s death); 9) victim is abducted or confined; 10) evidence of ritualism in the offense (i.e., was coded as present if the course of the offense clearly followed some type of script or role-play. For example, the presence of specific costumes, unusual paraphernalia suggesting the presence of script or role-play, see Nitschke et al., 2009); and 11) insertion of objects into victim’s body orifices (i.e., antemortem). Total scores can range from 0 to a maximum of 11; with a cut-off score of 4 or above indicating the probable presence of sexual sadism (Nitschke et al., 2009).

Individuals included in our study are only those presenting a score of at least 4 (see Nitschke et al., 2009). These individuals (i.e., both targeting children and adults) present an average score of 4.53 (*SD* = 0.83). Specifically, individuals involved in sadistic sexual crimes of children present an average score of 4.51 (*SD* = 0.76), while those involved in sadistic sexual crimes of adults present an average score of 4.53 (*SD* = 0.85). We observed a good reliability for the SeSaS items used to assess sexual sadism (Cronbach’s α = 0.84), which is congruent with recent studies (e.g., Gonçalves et al., 2020, α = 0.72; Oswald et al., 2019, α = 0.86). Finally, factor loading values are 0.5 or higher which suggests a satisfying relationship between the SeSaS items.

### Measures

#### Dependent variable

The dependent variable of the current study is a dichotomous variable. This variable describes categories to which the victims belong to (0 = *adult victims*; 1 = *child victims*).

#### Independent variables

We used 18 independent variables based on studies examining the crime-commission process of sadistic offenders that showed that these individuals followed specific steps based on the identification of vulnerable targets (Beauregard & Martineau, 2016; Burgess et al., 1986; Chopin & Beauregard, 2022; Dietz et al., 1990; Douglas et al., 2013), the commission of specific sexual and violent acts (Chopin & Beauregard, 2022; Dietz et al., 1990; Hazelwood et al., 1992; Healey et al., 2013; Warren et al., 1996), and finally the objective to avoid police detection (Beauregard & Martineau, 2016; Chopin et al., 2020; Reale et al., 2017, 2020). In order to see if there are differences between cases involving child and adult victims, we added a set of variables related to premeditation, victim characteristics, and routine victim activities to better understand the victim identification context. Additionally, we added variables related to the acts of violence (i.e., both sexual and non-sexual) perpetrated. This includes the following variables: 1) targeted by offenders (i.e., victim specifically targeted by the offender for his specific characteristics), 2) offender targeted a female victim, 3) victim was involved in domestic activities (e.g., watching TV, cooking), 4) victim was walking from a point to another, 5) victim was in a vehicle (e.g., travelling in a vehicle), 6) victim was involved in sports/outdoor activities, 7) victim was involved in social activities (e.g., visiting someone, partying, drinking in a bar), 9) offender and victim were strangers (i.e., did know each other before the assault). As to the sexual and non-sexual behaviors, we used the following variables: 10) sexual penetration (i.e., vaginal and/or anal penetration), 11) fellatio (i.e., offender forced the victim to perform a fellatio), 12) digital penetration (i.e., vaginal and/or anal), 13) masturbation (i.e., offender masturbated himself), 14) fondling, 15) offender had a weapon (i.e., does not imply that he used the weapon against the victim). As to the post-crime behaviors, we used the following variables: 16) offender killed the victim, 17) offender destroyed forensic evidence, and 18) offender protected his identity.

Previous studies found that sadistic crimes involve specific offender characteristics (Beauregard & Proulx, 2002; Brittain, 1970; Dietz et al., 1990; Martens, 2011) in comparison to other sexual crimes. To identify the specificities of individuals involved in sadistic crimes against children we used the following variables: 1) offender age, 2) offender was single, 3) offender living alone, 4) offender abused alcohol (i.e., diagnosis or reporting of a persistent alcohol abuse disorder), 5) offender abused drugs (i.e., diagnosis or reporting of a persistent drug abuse disorder), 6) offender had an active social life (i.e., participates in social situations and attends events where other people, including acquaintances and strangers, gather), 7) offender was socially isolated (i.e., a loner lifestyle with few social interactions), 8) offender had previous criminal convictions (i.e., general recidivism), 9) offender presented other paraphilic behaviors than sadism (i.e., fetishism, masochism, transvestism, exhibitionism, voyeurism, pedophilic sexual interests), 10) offender experienced sexual dysfunction (i.e., erectile and/or ejaculatory dysfunctions).

Finally, to explore manifestations of sadism, we used the eleven SeSaS items that we coded dichotomously (0 = *absent*, 1 = *present*).

### Analytical Strategies

This study follows a three-step process. The first level of analysis consists of assessing at the bivariate level (χ^2^) of analysis if the two groups (child vs. adult victims) differ significantly across the different phase of the crime-commission process as well as for the offender characteristics. The second level of analysis consists of integrating the significant dependent variables (see Tabachnick & Fidell, 2019) at the bivariate level in a sequential logistic regression. Such analytical process allows to better understand the impact of each variable while considering the other significant variable in the model. The sequencing of the logistic regression allows to identify which of the theoretical group of variables (i.e., crime-commission process characteristics and offender characteristics) is important to explain the variance of the dependent variable. Model 1 includes only the crime-commission process variables. Model 2 includes only the offender characteristics. Model 3 includes all two groups of variables. Multicollinearity was checked for the variables included in the multivariate analyses and no VIFs were above 1.174 and tolerance not below 0.852 (Appendix 1).

To determine whether sadistic sexual fantasies manifest themselves differently when the victims are children or adults, we performed multidimensional scaling analysis (MDS). The dimensions of sadism are combinations of several fantasies that are expressed in specific behaviors (Chopin & Beauregard, 2023). Therefore, in order to be able to compare how the manifestations combine with each other, we ran two MDS models (i.e., Model 1 corresponds to child victims; Model 2 corresponds to adult victims). The use of MDS is appropriate to identify the structure in a set of distance measures between a single set of objects or cases. Specifically, observations are assigned to specific locations in a conceptual low-dimensional space so that the distances between points in the space match the given similarities and dissimilarities as closely as possible (Giguère, 2006; Jaworska & Chupetlovska-Anastasova, 2009). The result is a least-squares representation of the objects in a low-dimensional space which improves the understanding of data structure. We used the Proximity Scaling (PROXCAL) procedure allowing to use dichotomous variables and to perform multidimensional scaling of proximity data to find a least-squares representation of the objects in a low-dimensional space (Busing et al., 1997) . In the current study we used only dichotomous variables allowing us to calculate Euclidean distances between the different indicators, while no specific standardized process was required. For each indicator, XY coordinates are assigned according to its proximity to the others. Such process allows representing the indicators graphically on a two-dimensional axis and to determine which dimensions they belong to, as well as their proximity to other indicators. To assess the PROXSCAL MDS model goodness of fit, we used the Normalized Raw Stress measure, the Dispersion Accounted For measure as well as the Tucker’s Coefficient of Congruence (Kruskal, 1964; Kruskal & Whish, 1978).

Ethical approval was obtained to conduct this research from the Institutional Review Board of the first, second, and fourth authors’ university.

## Results

Table 1 presents findings on the bivariate comparisons between sadistic crimes involving adult victims compared to child victims. Bivariate findings suggest that individuals who perpetrated sadistic crimes against children less often target female victims (χ^2^ = 20.00, φ = −0.19, *p* < .001), that were in vehicle/hitchhiking (χ^2^ = 4.72, φ = −0.09, *p* = .030), or who knew them (χ^2^ = 10.71, φ = −0.14, *p* = .001) compared to individuals with adult victims. These individuals were more likely to masturbate (χ ^2^ = 29.78, φ = 0.23, *p* < .001) during the assault, and to engage in fondling (χ ^2^ = 10.23, φ = 0.14, *p* = .001). Individuals who committed sadistic crimes against children were less likely to use a weapon (χ^2^ = 15.00, φ = −0.17, *p* < .001). As to the post-crime behaviors, individuals who perpetrated sadistic crimes against children less often protected their identity (χ^2^ = 17.14, φ = −0.18, *p* < .001), and destroyed forensic evidence (χ^2^ = 5.53, φ = −0.10, *p* = .019). These individuals were less likely to abuse alcohol (χ^2^ = 11.98, φ = −0.15, *p* = .001), and to experience sexual dysfunctions (χ ^2^ = 4.61, φ = −0.09, *p* < .001). Finally, individuals who committed sadistic crimes against children were more likely to present other paraphilic behaviors than sadism (χ^2^ = 4.61, φ = −0.09, *p* < .001).

**Table 1.** Bivariate Statistics of the Crime and Offender Characteristics According to the Age of victims (*N* = 544).

	Adult victims *n* = 443		Child victims *n* = 101		ANOVA/χ^2^	φ/ω^2^
	*n*	%	*n*	%		
Target selection						
Targeted by offenders	166	37.47	46	45.54	2.25	0.06
Offender targeted a female victim	414	93.45	80	79.21	20.00***	−0.19
Victim was involved in home activities	118	26.64	21	20.79	1.48	−0.05
Victim was walking from a point to another	147	33.18	40	39.60	1.50	0.05
Victim was in vehicle/hitchhiking	49	11.06	4	3.96	4.72*	−0.09
Victim was involved in sports/outdoor activities	7	1.58	4	3.96	2.35	0.07
Victim was involved in social activities	54	12.19	18	17.82	2.27	0.07
Offender and victim were strangers	308	69.53	53	52.48	10.71***	−0.14
Sexual and non-sexual behaviors						
Sexual penetration	357	80.59	81	80.20	0.01	0.00
Fellatio	253	57.11	64	63.37	1.32	0.05
Digital penetration	144	32.51	41	40.59	2.40	0.07
Masturbation	84	18.96	45	44.55	29.78***	0.23
Fondling	177	39.95	58	57.43	10.23**	0.14
Offender used weapon	226	51.02	30	29.70	15.00***	−0.17
Post crime behaviors		0.00		0.00		
Offender killed the victim	73	16.48	12	11.88	1.32	−0.49
Offender destroyed forensic evidence	88	19.86	10	9.90	5.53*	−0.10
Offender protected identity	155	34.99	14	13.86	17.14***	−0.18
Offender characteristics						
Offender age	31.03 (*SD* = 10.12)		31.17 (*SD* = 11.86)		1.54^a^	0.00^b^
Offender was single	229	51.69	54	53.47	0.10	0.01
Offender living alone	82	18.51	24	23.76	1.45	0.05
Offender abused alcohol	178	40.18	22	21.78	11.98***	−0.15
Offender abused drugs	94	21.22	14	13.86	2.80	−0.07
Offender had an active social life	23	5.19	5	4.95	0.01	0.00
Offender had loner disorders	49	11.06	17	16.83	2.57	0.07
Offender had previous criminal conviction	95	21.44	14	13.86	2.95	−0.07
Offender had other paraphilic behaviors than sadism	133	30.02	54	53.47	20.04***	0.19
Offender had sexual dysfunction	115	25.96	16	15.84	4.61*	−0.09

a Corresponds to the ANOVA.

b Corresponds to the omega-squared.

* *p* < .05. **p < .01. ***p < .001.

Table 2 presents findings of the sequential logistic regression examining factors associated with the type of victims in sadistic sexual crimes. Model 1 includes only the variables related to the crime-commission process. This model presents an area under the curve (AUC) of 0.782. Findings show that offenders targeting female victims (β = −1.07, *p* = .002), that were in a vehicle/hitchhiking (β = −1.04, *p* = .023), who used weapon (β = −0.79, *p* = .002), and who used strategies to protect their identity (β = −1.00, *p* = .003) were less likely to perpetrate sadistic crimes against children. Individuals who masturbate during sadistic assaults were more likely to target children (β = 1.25, *p* < .001). Model 2 includes only the variables related to the crime-commission process and presents an AUC of 0.701. Individuals who abused alcohol (β = −0.89, *p* = .001), and who experienced sexual dysfunctions (β = −0.70, *p* = .020) were less likely to perpetrate sadistic crimes against children. Offenders presenting other paraphilias than sadism were more likely to target children (β = 1.04, *p* < .001). Model 3 considers crime-commission process and offender characteristics variables and presents an AUC of 0.802. Individuals targeting female victims (β = −0.90, *p* = .019), who were in vehicle or hitchhiking (β = −1.19, *p* = .037), and who used a weapon (β = −0.83, *p* = .002) were less likely to perpetrate sadistic crimes against children. Sadistic offenders who used strategies to protect their identity (β = −1.14, *p* = .001), who had problems of alcohol abuse (β = −1.04, *p* < .001) as well as sexual dysfunctions (β = −0.74, *p* = .028) were less likely to target child victims. However, cases where offenders masturbated during the assault were more likely to involved child victims (β = 1.08, *p* = .029).

**Table 2.** Logistic Sequential Regression of Factors Associated With Sadistic Sexual Offending of Children (*N* = 544).

	Model 1			Model 2					
	β	*SE*	Exp(β)	β	*SE*	Exp(β)	β	*SE*	Exp(β)
Offender targeted a female victim	−1.07	0.35	0.34***				−0.90	0.38	0.41*
Victim was in vehicle/hitchhiking	−1.04	0.56	0.36*				−1.19	0.57	0.31*
Offender and victim were strangers	−0.32	0.25	0.73				−0.31	0.27	0.73
Masturbation	1.25	0.27	3.49***				1.08	0.29	2.96***
Fondling	0.44	0.25	1.56				0.45	0.26	1.57
Offender used weapon	−0.79	0.26	0.45**				−0.83	0.27	0.44**
Offender destroyed forensic evidence	−0.75	0.39	0.47				−0.72	0.41	0.49
Offender protected identity	−1.00	0.33	0.37**				−1.14	0.34	0.32**
Offender abused alcohol				−0.89	0.27**	0.41	−1.04	0.29	0.36***
Offender had other paraphilia than sadism				1.04	0.23***	2.83	0.45	0.27	1.56*
Offender had sexual dysfunction				−0.70	0.30*	0.50	−0.74	0.34	0.48*
Constant	−0.22	0.37***	0.81	−1.49	0.18***	0.23	0.04	0.46	1.04
χ^2^	85.74***			37.9			108.72***		
−2 Log Likelihood	436.359			484.19			413.38		
Hosmer and Lemeshow test	4.92			2.74			5.21		
AUC	0.782			0.701			0.802		

* *p* < .05. **p < .01. ***p < .001

Table 3 presents goodness of fit stress and fit measures of the PROXSCAL MDS analysis. The measure-of-fit for this solution normalized raw STRESS produces 0.05 (Model 1) and 0.04 (Model 2) values. This coefficient varies from 0 to 1 and should be less than 0.05 as a good fit, which is the case for both models (Kruskal, 1964; Kruskal & Whish, 1978). The Tucker’s φ Coefficient of Congruence indicates that 97% of the variance in the Model 1 and 97% in the Model 2 are explained by two dimensions. This coefficient should be ideally more than 90% to confirm that the two-dimension representation is appropriate for the data used (Jensen, 1998; Lorenzo-Seva & Ten Berge, 2006), which is the case here.

**Table 3.** PROXSCAL MDS Goodness of Fit Stress and Fit Measures.

	Model 1	Model 2
Normalized raw stress	0.05	0.04
Dispersion accounted for	0.94	0.95
Tucker’s φ coefficient of congruence	0.97	0.97

*Note*. Model 1 corresponds to the child victims model. Model 2 corresponds to the adult victims model.

**Figure 1.**
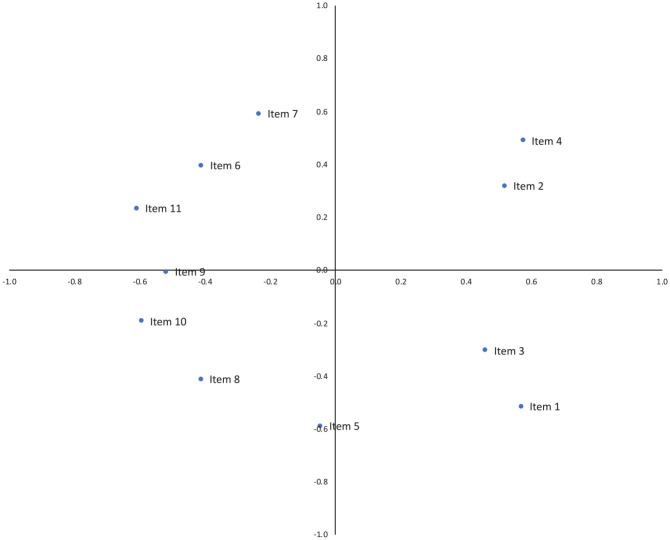
PROXSCAL MDS plot of Model 1 (child victims).

**Figure 2.**
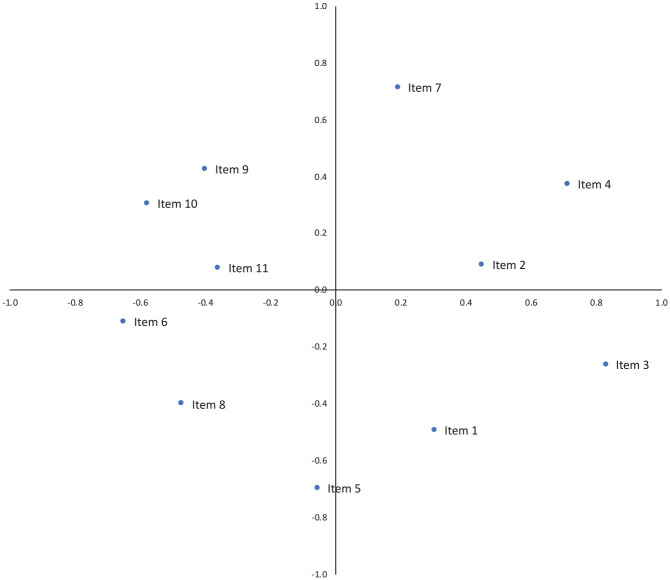
PROXSCAL MDS plot of Model 2 (adult victims).

Table 4 and Figures 1 and 2 present the proximity coordinates of the PROXSCAL MDS Model 1 and 2. The descriptive analysis of SeSaS items distribution between cases involving child and adult victims are provided in Appendix 2. The model 1 corresponds to the distribution of sadism manifestations in a two-dimensional space for sadistic crimes against children. The results suggest four distinct patterns (numbers in brackets are proximities coordinates). In the first category, we observe an association between Item 4 (sexually aroused, 0.57; 0.48) and Item 2 (power/domination, 0.51; −0.31). The second pattern suggests an association between Item 3 (humiliation; 0.46; 0.30) and Item 1 (gratuitous violence; 0.56; 0.51). A third pattern shows the association between Item 10 (mutilation of non-sexual parts; −0.59; −0.19) and Item 8 (foreign object insertion, −0.41; −0.41). We observe that Item 5 (torture, −0.04; −0.59) is at the intersection between these two patterns. This suggests that it is associated with both of these items. Finally, the last pattern suggests an association between Item 11 (trophies, −0.60; −0.23), Item 6 (ritualism, −0.41; 0.39), and Item 7 (abduction, 0.03; 0.59). We observe that Item 9 (mutilation of sexual parts, −0.51; −0.07) is at the intersection between these two patterns. This suggests that it is associated with both of these items.

**Table 4.** PROXSCAL MDS Proximities Coordinates (*N* = 544).

	Model 1 child victims			Model 2 adult victims		
	%	1	2	%	1	2
Item 1. Offender engages in gratuitous violence toward or wounding of the victim	49.55	0.57	−0.52	71.56	0.31	−0.49
Item 2. Offender exercises power/control/domination over the victim	57.42	0.52	0.32	62.53	0.45	0.09
Item 3. Offender humiliates/or degrades the victim	97.03	0.46	−0.30	98.20	0.83	−0.26
Item 4. Offender is sexually aroused by the act	69.31	0.58	0.49	71.78	0.72	0.37
Item 5. Offender tortures the victim or engages in acts of cruelty to the victim	44.55	−0.05	−0.59	41.98	−0.05	−0.70
Item 6. Evidence of ritualism in offense	21.78	−0.41	0.39	17.15	−0.65	−0.11
Item 7. Victim is abducted/or confined	50.49	−0.03	0.59	35.89	0.19	0.71
Item 8. Insertion of objects into victim’s bodily orifice	30.69	−0.41	−0.41	22.35	−0.47	−0.40
Item 9. Offender Mutilates sexual parts of the victim’s body	4.95	−0.52	−0.01	6.99	−0.40	0.42
Item 10. Offender mutilates nonsexual parts of the victim’s body	11.88	−0.59	−0.19	12.41	−0.58	0.30
Item 11. Offender keeps trophies	13.86	−0.61	0.23	11.96	−0.36	0.08

*Note*. Model 1 corresponds to the child victim model. Model 2 corresponds to the adult victim model

Model 2 corresponds to the distribution of sadism manifestations in a two-dimensional space for sadistic crimes against adult victims. The first pattern suggests an association between Item 7 (abduction, 0.19; 0.71), Item 4 (sexually aroused, 0.71; 0.37), and Item 2 (power/domination, 0.45; 0.08). The second pattern shows the association between Item 3 (humiliation, 0.83; −0.26) and Item 1 (gratuitous violence, 0.30; −0.49). The third pattern suggests an association between Item 8 (foreign object insertion, −0.46; −0.40) and Item 6 (ritualism, −0.64; −0.11). We observe that Item 5 (torture, −0.05; −0.69) is at the intersection between these two patterns. This suggests that it is associated with both of these items. Finally, the last pattern suggests an association between Item 11 (trophies, −0.35; −0.07), Item 10 (mutilation of non-sexual parts, −0.57; −0.30), and Item 9 (mutilation of sexual parts, −0.39; 0.42).

## Discussion

This study explored the differences in the characteristics, crime-commission process, and the dimensions of sadism between sadistic crimes of children compared to those of adults. In terms of offender characteristics, those who commit sadistic sexual offenses against adults compared to child victims overlap considerably, however, there are some noteworthy differences. First, we find that sadistic offenders with child victims are less likely to abuse alcohol prior to the crime. It is possible that the use of alcohol is less common in sadistic crimes involving children because it less compatible with their crime script. For example, the use of alcohol is associated with higher levels of victim injury (Abbey et al., 2002; Brecklin & Ullman, 2010; Chopin & Beauregard, 2019), which is consistent with the higher frequency of gratuitous violence in our sample of sadistic crimes of adult victims (71.5%) compared to child victims (49.5%). Interestingly we also find perpetrators of sadistic sexual offenses against children are more likely to have male victims and masturbate at the crime scene. Individuals who sexually offend against male victims are more likely to exhibit pedophilia (e.g., Levenson & Morin, 2006) and masturbation during the offense is consistent with the crime-commission process of pedophilic child molesters (e.g., Beauregard et al., 2008b). Thus, it is possible to hypothesize that some offenders in the current study are influenced to offend as a result of both sexually sadistic and pedophilic sexual preferences. This is consistent with our finding that offenders with child victims were also more likely to present with an additional paraphilia.

There are also several noteworthy distinctions in the crime-commission process between sadistic crimes of children and adults. For example, we find that sadistic offenders with adult victims are more likely to target a victim walking/hitchhiking, to bring a weapon to the offense, to protect their identity and destroy and remove evidence as a strategy to reduce their risk of apprehension. A more structured and organized crime-commission process is consistent with sadistic crimes described in prior literature and is thought to reflect their deviant sexual fantasies (e.g., Beauregard & Proulx, 2002; Dietz et al., 1990; Reale et al., 2017, 2020; Warren et al., 1996). Although it is not entirely surprising that offenders who engage in sadistic crimes against adults are more likely to bring a weapon to the offense, it points to important differences between the two groups. Bringing a weapon to the offense suggests structured premeditation, in that they were preparing to overcome victim resistance and/or use the weapon to maintain victim compliance (Rossmo, 2000). Conversely, it appears that offenders with child victims may not prepare for a violent encounter because they anticipate a child victim will be easier to control and less likely to report/identify the offender post-crime. This suggests that the choice of an adult or child victim is not randomly selected but related to sexual preferences and underlying deviant sexual fantasies.

In terms of differences in behavioral manifestations of sadism between child and adult, we observed that there are two dimensions that are stable (Items 3/1) and (Items 4/2) across both groups. More specifically, we observed that both adult and child sadistic offenses overlap in the areas of violence and humiliation, as well as sexual arousal and power and control. These are consistent with the key distinguishing features of sadism, which are thought to be humiliation, degradation, subjugation, and suffering of the victim, which makes the sadist feel powerful and sexually aroused (e.g., Chopin & Beauregard, 2023; Healey et al., 2013; Longpré et al. 2019). This suggests that the core behavioral indicators of sexual sadism are similar between both victim age groups. Interestingly, however, the violence and humiliation dimension in the current sample of child victims contrasts with Chopin and Beauregard (2022), who found that humiliation and degradation was totally absent in their sample of sadistic child sexual homicide offenders. The differences between these two samples may be related to our study including both sexual assault and sexual homicide whereas Chopin and Beauregard (2022) exclusively examined homicide. For example, humiliation may be particularly difficult to ascertain in cases of sadistic sexual homicide where the experience of the victim is not available. Additionally, Chopin and Beauregard (2022) assessed sadism using a different tool specific to sexual homicide (i.e., the SADSEX-SH), while we used the SeSas, which may also explain why the operationalized of some sadistic behaviors differed between the two samples.

We also found a dimension specific to child victims involving the collection of trophies or souvenirs, ritualism, and confinement/abduction (Items 11, 6, and 7) that was not observed among cases with adult victims. This is similar to the ritualism and/or offense planning dimension found in Longpré et al. (2019), who also examined sadistic crimes of children and adults, although no comparisons were made between the two samples. This suggests that some offenders with child victims are particularly oriented towards behaviors that allow them to act out on their deviant sexual fantasies, both during (i.e., confinement and ritualism) and after the crime (i.e., through souvenirs or trophies) to achieve sexual satisfaction. This is similar to the *sadistic-muted* type of child molester (Knight et al., 1989) which was also characterized by behavioral evidence of sadistic sexual fantasy and less severe acts of violence.

Lastly, there was a fourth dimension for child victims involving more severe forms of violence, including mutilation (Items 9/10), foreign object insertion (Item 8) and torture (Item 5). This subgroup overlaps considerably with sadistic behaviors associated with the most extreme form of sexual violence (i.e., sexual homicide) and share similarities to the *sadistic high-injury type* observed by Knight et al. (1989). Moreover, sexual mutilation and foreign object insertion are markers of extreme sexual sadism and sadistic sexual murder (Healey et al., 2013; Longpré et al., 2020; Mokros et al., 2012; Nitschke et al., 2009). Thus, individuals who commit these crimes might need extreme forms of physical violence to obtain sexual gratification.

For the adult victims, there were two distinct dimensions that reflect the notions of torturing and suffering, with one that is more sexualized through foreign object insertion and torture (Items 8 and 5) while the other is more oriented towards mutilation (Items 9, 10). Taken together, this suggests that some sadistic crimes of adult victims appear to reflect fantasies that are more oriented towards extreme behaviors and the physical suffering of the victim to achieve sexual satisfaction. This builds on previous evidence that as sadistic fantasies vary, so does the degree of violence (e.g., Chopin & Beauregard, 2022; Warren et al., 1996).

There are also some limitations to the current study that should be noted. Firstly, limitations exist regarding the use of police data in terms of validity and reliability. For example, this research is concerned only with cases that have been reported to the authorities and we cannot generalize to all cases of sadistic crimes of adults and child victims. Moreover, our study focuses only on solved cases, and we cannot exclude the possibility that unsolved cases may represent distinctive patterns. Further, we only assessed sexual sadism using the SeSaS scale and could not corroborate with a clinical diagnosis. Even though crime scene behaviors are considered to be the most valid method to assess sadism (Longpré et al., 2019), there is a possibility that some cases were not identified and consequently not included in our sample. Moreover, although all offenders in the sample scored at least a 4 on the SeSas, it is possible that some offenders were miscategorized. For example, some offenders who engage in a high degree of violence and harm against a victim are motivated by anger and not sexual pleasure (Higgs et al., 2017).

## Implications/Conclusion

The purpose of the present study was to examine whether sadistic crimes of children differ from sadistic sexual crimes of adults in their lifestyle, modus operandi (pre-crime, crime, post-crime) and sexual sadism (manifestations). We found that sadistic fantasies manifested into four dimensions for both adult and child victims, which is are consistent with existing literature that shows that variations in the deviant fantasies influence the offender’s behaviors throughout the crime-commission process (e.g., Chopin & Beauregard, 2023, 2022; Knight et al., 1989; Longpré et al., 2018, 2019; Reale et al., 2017, 2020). For example, a key difference between these two groups is that some sadistic sexual offenses involving children appear to be reflective of deviant fantasies related to overlapping paraphilias (e.g., sadism and pedophilia). Conversely, sadistic crimes involving adult victims involve a crime-commission process that involves a greater degree of structure to control their adult victim and decrease their risk of identification. This more versatile offense behaviors in cases with adult victims is consistent with the notion of generality among individuals who perpetrate sexual offenses (e.g., Lussier, 2005).

Given the lack of research on sadistic crimes of children, our findings help to provide validity to the use of behavioural indicators of sexual sadism as an additional assessment tool in this population. For example, our findings indicate that assessing treatment needs and recidivism risks for individuals who commit sadistic sexual crimes against children will need to account for potential overlapping paraphilias (e.g., pedophilia) that relate to underlying deviant fantasies and motivations for offending. We also identified a dimension in the adult victim subgroup associated with sexual and non-sexual mutilation as well as foreign object insertion and a dimension in the child victim subgroup associated with sexual and non-sexual mutilation. Nitschke et al. (2009) and Mokros et al. (2012) both indicate that these behaviors represent more extreme and uncommon expressions of sexual sadism. This is especially important as sadism is associated with the most severe forms of offending, including sexual homicide (e.g., Brittain, 1970; Dietz et al., 1990). For instance, Chopin et al. (2022) found that foreign object insertion and mutilation (both sexual and non-sexual) increased the likelihood of a lethal outcome occurring. By understanding the different behavioral manifestations of sadism, practitioners may also be able to identify those whose expression of sadism is associated with the more severe end of the sadistic continuum. These indicators may be particularly important for clinicians to consider, especially when the individual is likely to deny or downplay sadistic urges or fantasies, such as in cases of sexual homicide (Stefanska et al., 2019). This latter point is also relevant for police investigations, as investigators are tasked with assessing the presence of sexual sadism through crime scene behaviours when perpetrators deny their underlying motivations (Hazelwood et al., 1992). Our findings show that the crime-commission process in sadistic sexual crimes can be influenced by the type of victim (i.e., child or adult), including the amount of force, sadistic acts, and level of detection avoidance strategies used. Thus, findings could aid in more accurately identifying whether a crime is indicative of sexual sadism (and its varying degrees) in cases that involve either adult or child victims.

## Appendix

**Appendix 1.** Collinearity Diagnostic (*n* = 544).

Variables	Tolerance	VIF
Offender targeted a female victim	0.919	1.088
Victim was in vehicle/hitchhiking	0.962	1.039
Offender and victim were strangers	0.852	1.174
Masturbation	0.857	1.166
Fondling	0.890	1.123
Offender used weapon	0.937	1.067
Offender destroyed forensic evidence	0.940	1.063
Offender protected identity	0.882	1.134
Offender abused alcohol	0.929	1.076
Offender had other paraphilia than sadism	0.866	1.155
Offender had sexual dysfunction	0.940	1.064

**Appendix 2.** Descriptive Analyses of SeSaS Items Distribution Between Cases Involving Child and Adult Victims (*N* = 544).

	Child victims		Adult victims		Total	
	%	*n*	%	*n*	%	*n*
Item 1. Offender engages in gratuitous violence toward or wounding of the victim	49.55	50	71.56	317	67.46	367
Item 2. Offender exercises power/control/domination over the victim	57.42	58	62.53	277	61.58	335
Item 3. Offender humiliates/or degrades the victim	97.03	98	98.2	345	81.43	443
Item 4. Offender is sexually aroused by the act	69.31	70	71.78	318	71.32	388
Item 5. Offender tortures the victim or engages in acts of cruelty to the victim	44.55	45	41.98	186	42.46	231
Item 6. Evidence of ritualism in offense	21.78	22	17.15	76	18.01	98
Item 7. Victim is abducted/or confined	50.49	51	35.89	159	38.60	210
Item 8. Insertion of objects into victim’s bodily orifice	30.69	31	22.35	99	23.90	130
Item 9. Offender Mutilates sexual parts of the victim’s body	4.95	5	6.99	31	6.62	36
Item 10. Offender mutilates nonsexual parts of the victim’s body	11.88	12	12.41	55	12.32	67
Item 11. Offender keeps trophies	13.86	14	11.96	53	12.32	67

## Notes

1. The SeSaS items were coded by the second author following the guidelines suggested by Nitschke et al. (2009).
